# Brave Editors

**Published:** 1871-07

**Authors:** 


### BRAVE EDITORS.

There are two brave men, morally brave, in the United States: one, the editor of the *Watchman and Reflector*, Boston, and the other of the *New York Evening Mail*, — men who are not afraid to speak their mind in the way of just criticism on all books and publications which they " notice." Irreligion, vice and crime, and indecency uniformly end in disease and destitution, and an early or premature death; and whatever books and magazines and newspapers send vicious reading and indecent narrations and blasphemous utterances into the families which patronize them, certainly merit a just criticism on the part of intelligent, judicious, and conscientious conductors of the periodical literature of the country.

The first named paper has for a number of years been a faithful *Watchman* in this connection, and has been fearless in its rebuke of publishers, however high their standing, who aid in flooding the nation with reading calculated to corrupt the morals of those into whose families they come. *The Evening Mail* of May 5th does not fear to call to account the two largest and wealthiest publishing houses in this country for publishing a serial " story " of a man who " has already written enough in it that is bad to blast forever his fame as a reputable writer, and gives deliberate hints of a sin so utterly evil and horrible as not to be named among Christians. Those who, as publishers, assist in this corruption of our homes have no right to think they can hide behind a hitherto great name, or that they are less responsible than the author before man and before God. If these most honorable members of our business class are thus to sacrifice principle, our nation is gone, for its morality is its life. If the police have a right to seize what with universal endorsement they have seized, why are they not at work here? The only distinction of this story seems to be that it is worse, because the author has skill to sweeten his poison, and because the imprint of its publishers, hitherto of the highest fame, allows it to be read openly, instead of clandestinely, in respectable houses. No man who desires the purity of his wife and of his children can take it home. And we must say plainly to those publishers that if they would not injure their good repute, as men and Christians, this story must be forthwith dropped from their columns, to be continued only in those of that weekly disgrace to our city, in which it has already found its proper place."

No Christian man can possibly read a recent Boston pictorial in which some machine-poetry is illustrated, the last two lines of which are nothing short of impious blasphemy. This publication also contains the indecent story about which the *Evening Mail* has written so scathingly; a paper which well merits the patronage not only of Christian families but of all good citizens, conducted as it has always been, with an ability and industry, and a watchfulness over its columns, which renders it one of the very best evening papers of this great city; and as such, it has found a way and a welcome into the very best families, where culture and refinement and purity meet in the same household, and gather evening after evening around the same hearth.

